# Chemically Responsive Hydrogel Deformation Mechanics: A Review

**DOI:** 10.3390/molecules24193521

**Published:** 2019-09-28

**Authors:** Eanna Fennell, Jacques M. Huyghe

**Affiliations:** 1 Bernal Institute, University of Limerick, V94 T9PX Limerick, Ireland; 2 School of Engineering, University of Limerick, V94 T9PX Limerick, Ireland; 3 Department of Mechanical Engineering, Technical University of Eindhoven, 5600 MB Eindhoven, The Netherlands

**Keywords:** hydrogels, superabsorbent polymers, osmotic swelling, finite deformation, kinetics, thermodynamics, hydrogel mechanics, surface instabilities, chemically-responsive

## Abstract

A hydrogel is a polymeric three-dimensional network structure. The applications of this material type are diversified over a broad range of fields. Their soft nature and similarity to natural tissue allows for their use in tissue engineering, medical devices, agriculture, and industrial health products. However, as the demand for such materials increases, the need to understand the material mechanics is paramount across all fields. As a result, many attempts to numerically model the swelling and drying of chemically responsive hydrogels have been published. Material characterization of the mechanical properties of a gel bead under osmotic loading is difficult. As a result, much of the literature has implemented variants of swelling theories. Therefore, this article focuses on reviewing the current literature and outlining the numerical models of swelling hydrogels as a result of exposure to chemical stimuli. Furthermore, the experimental techniques attempting to quantify bulk gel mechanics are summarized. Finally, an overview on the mechanisms governing the formation of geometric surface instabilities during transient swelling of soft materials is provided.

## 1. Introduction

**Figure 1:**
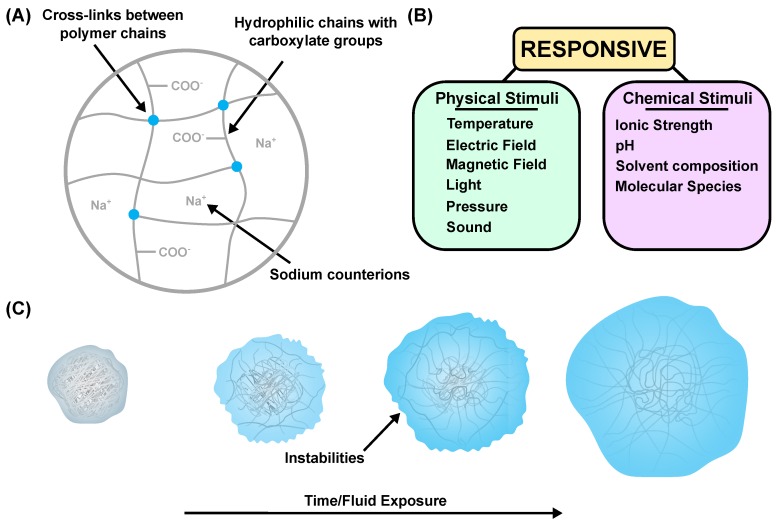
(**A**) Material structure of a superabsorbent polymer (sodium neutralized polyacrylic acid). (**B**) Response classification of hydrogels, separated into physically and chemically responsive gels and including the stimuli for each. (**C**) Schematic of dry gel (left) exposed to a swelling solution, showing the transience of the process (including surface instabilities) and finally coming to equilibrium (right).

A hydrogel is a three-dimensional network structure composed of cross-linked polymer chains (see Figure 1A), which has the ability to absorb a large volume of solution. This can be attributed to hydrophilic residues, which are found on the monomer chains or as lateral terminal chains, or to counter-ions in the network, resulting in an associated osmotic pressure. The cross-links resist the deformation caused by fluid absorption and stop the material from dissolving into the swelling solution. Hydrogels can either be synthetic or natural. Natural gels, such as gelatine, agar, polysaccharides, bacterial biofilms, and extra-cellular matrices, can be found throughout all areas of life and nature [1,2]. However, synthetic polymers have only existed since the 1960s [3]. Since then, the field of synthetic hydrogels has exploded, with the number of publications on the topic increasing each year. As a result of the ability to tailor synthetic polymers to a given application, they have often replaced natural polymers in academia and industry [4]. Hydrogels are generally created through a process known as gelation. This process consists of the linking of macro-molecular monomer chains in order to create progressively larger branched chains. The framework of these polydisperse soluble branched chains is called ‘sol’. The linking process continues to increase the size of the network with decreasing solubility to form a polymer with finite branched chains. This formation to a structured network is known as the ‘sol–gel’ transition [5]. Gelation can be classed into two separate categories—physical or chemical—depending on the type of interactions. Chemical gelation refers to the formation of covalent bonds between the branched chains and always results in a strong gel (i.e., permanent and irreversible). Physical gelation can result in either a strong or weak gel, and consists of physical bonds (i.e., interactions such as ionic, polar, or induced dipole interactions) between the polymer chains [6]. A particular type of hydrogel, known as superabsorbent polymers (SAP), has the ability to swell up to 100 times its original volume [7]. These structures are typically synthesized through the radical polymerization of monomers. Chemical cross-linking of these monomer chains is implemented through co-polymerization of multi-functional monomers into the network [8]. This review will consider both chemically and physically cross-linked gels.

Hydrogels can be grouped into various material classifications, such as by preparation techniques, cross-linking type, degradability, and response to name a few [1,4,9]. An in-depth review of each of the various classification groups is outside the scope of this article; however, Figure 1B summarizes the ‘response’ class of hydrogels. These gels respond mechanically to either chemical or physical stimuli. The chemically responsive class responds to changes in ionic strength, pH, composition, and molecular species in the external solution/environment (many SAPs are chemically responsive hydrogels). This response is generally a swelling or shrinking of the material (i.e., a volumetric change), also called deformation. Many attempts to numerically replicate such deformation have been published [10,11]. However, due to the difficulty of mechanically characterizing the strains of the transient swelling, there exist a wide variety in the theories and constitutive models which have been implemented. Furthermore, during transient swelling, the surface of the gel is often observed to buckle and form a wrinkling pattern. Again, experimentally characterizing this phenomenon quantitatively is difficult, as a result of its seemingly random nature.

The ability to influence the characteristics of hydrogels allows for a broad range of applications which are extensive across many diverse fields [12]. They are used widely in drug delivery systems and tissue engineering scaffolds in medicine, as well as being key in the design of smart biodevices and biosensors [13,14]. In agriculture, they have been studied for use in moisture retention in soil and have also been implemented in various hygiene products (especially superabsorbents) [8,15,16]. As the prerequisite of these applications is to control the rate and magnitude of swelling, an understanding of gel mechanics at large deformation is paramount in ensuring the efficacy of the products.

Therefore, this article focuses on reviewing the current literature surrounding the deformation mechanics of chemically responsive hydrogels. This includes the computational models of hydrogel free swelling, as well as the experimental techniques used to quantify gel deformation. A review on the mechanisms governing surface instabilities during transient swelling will also be conducted.

## 2. Hydrogel Swelling Theory

Generally, chemically responsive hydrogel swelling, especially for superabsorbent polymers, is triggered by two mechanisms. First, when the system is exposed to an aqueous solution, water molecules are attracted to the polymer chains across the gel surface in a process known as mixing. Secondly, a Donnan osmotic pressure is generated by the difference between the concentration of counter-ions inside the gel and concentration of an ionic compound in the external aqueous solution. As a result, fluid moves across the semi-permeable gel boundary. The direction of fluid flow depends on the sign of the sum of the osmotic pressure difference and mixing pressures. If the chemical potential of the fluid outside is greater than inside, the gel swells; on the other hand, if the chemical potential of the fluid inside is greater than outside, the gel shrinks. The magnitude of swelling or shrinking is a function of the size of the osmotic pressure difference, the mixing pressure, and the stiffness of the macro-molecular network. The ability to theoretically model the interaction between this osmotic pressure difference, the mixing pressure, and its effect on the deformation of the gel is key in implementing an accurate numerical model.

As with all theoretical models, assumptions about the system have to be made. Here, an ideal elastomeric gel is taken to have two main assumptions: The first assumption, known as molecular incompressibility, states that the volume of a gel system is taken as the sum of the dry gel volume and the absorbed solvent volume (Equation (1)) [17]. The second assumption, known as the Frenkel–Flory–Rehner hypothesis, states that the Helmholtz free energy of a hydrogel system is the sum of three parts: The energy of the mixing of the solvent and the polymer ($F_{mixing}$), the energy associated with the elastic stretching of solid matrix ($F_{elastic}$), and finally the energy of the ionic osmosis ($F_{ion}$) (Equation (2)) [18,19,20,21,22].

(1) $$\sum_{\alpha =s,p,+,-}\phi_{\alpha }=1,$$

(2) $$\Delta F_{total}=\Delta F_{mixing}+\Delta F_{elastic}+\Delta F_{ionic},$$

where $\phi_{\alpha }$ is the volume fraction of the constituent α (where α is s for solvent, p for polymer, + for positive ions, and − for negative ions). Several studies have shown that the perfect seperability of the energies defined by the Flory–Frenkel–Rehner (Equation (2)) theory is not exact [23,24,25]. However, an alternative to separating the free energies of the system has not been published and, therefore, for the purpose of the computational models outlined below, Equation (2) is assumed to be true [21].

The mixing and ionic energy terms are the driving forces of gel swelling, while the elastic term restricts the gel from extending indefinitely and eventually balances the swelling terms to allow the gel to reach equilibrium. The driving forces of the swelling ($\Pi_{swelling}$) can then be denoted by their osmotic pressures (Equation (3)):

(3) $$\Pi_{swelling}=\Pi_{ionic}+\Pi_{mixing}.$$

There have been many published theories coupling the chemical forces to the mechanical response of gels [26,27]. Generally, they draw upon two different research traditions: Flory–Rehner theory and mixture theory. Flory–Rehner theory is more concerned with the micro-structure of the gel and how individual polymer chains effect the system, whereas mixture theory assumes the continuum of each constituent of the mixture is always occupied at every point in the system. Therefore, mixture theory has more of an emphasis on the macro-structure of the gel and tends to average out the micro-structure considerably more than Flory–Rehner theory. Hence, the elastic energy of Flory–Rehner theory is described by statistical mechanics, while mixture theory is described by continuum mechanics. These two frameworks are similar in their use of the swelling pressures ($\Pi_{ion}$ and $\Pi_{mix}$), but differ substantially in their theoretical description of the elastic energy.

### 2.1. Mixing Energy

When a hydrophilic polymer comes into contact with an aqueous solution, the solvent molecules are attracted towards the polymer through a weak van der Waals force in a process called mixing (i.e., mixing of polymer and solution). The energy associated with the mixing of this solution–polymer blend can be described by the Flory–Huggins lattice theory of polymer solutions [28,29]. This theory assumes that the total number of lattice sites in a rigid lattice frame ($N_{0}$) are comprised of distributed solvent molecules ($N_{s}$) and repeat polymer units ($N_{p}r$):

(4) $$N_{0}=N_{s}+N_{p}r.$$

We define the Flory–Huggins approximation of the entropy ($\Delta s_{mix}$) associated with the mixing of polymer solution as

(5) $$\Delta s_{mix}=\frac{\Delta S_{mix}}{N_{0}}\approx -k_{B}\left[\frac{\phi_{p}}{\left(r.v_{r}\right)}ln\left(\phi_{p}\right)+\frac{\phi_{s}}{v_{s}}ln\left(\phi_{s}\right)\right],$$

where $k_{B}$ is the Boltzmann constant and $v_{r,s}$ are the volumes of a repeat polymer unit and a solvent molecule, respectively. The volume fractions of solvent (*s*) and polymer (*p*) are denoted as

(6) $$\phi_{s}=\frac{N_{s}v_{s}}{\left(N_{s}v_{s}+N_{p}rv_{r}\right)},$$

(7) $$\phi_{p}=\frac{N_{p}v_{p}}{\left(N_{s}v_{s}+N_{p}rv_{r}\right)}.$$

The Gibbs free energy associated with mixing ($\Delta G_{mix}$) depends on both the entropy ($\Delta S_{mix}$) and the internal energy associated with the enthalpy ($\Delta U_{mix}$), taking into account that the polymer solution mixing can either be an exothermic or endothermic reaction:

(8) $$\Delta G_{mix}\approx \Delta F_{mix}=\Delta U_{mix}-T\Delta S_{mix}.$$

When the volume change from the mixing of polymer and solvent is negligible, the Gibbs free energy can be approximated as the Helmholtz free energy ($\Delta F_{mix}$). So far, the solvent and polymer terms have been written separately but, in order to take into account the actual mixing of the two, an interaction parameter must be defined. This is called the Flory–Huggins interaction parameter, χ, which is related to the enthalpy in the mixture by

(9) $$\chi_{p,s}=\frac{\Delta U_{mix}}{k_{B}TN_{s}\phi_{p}}.$$

**Figure 2:**
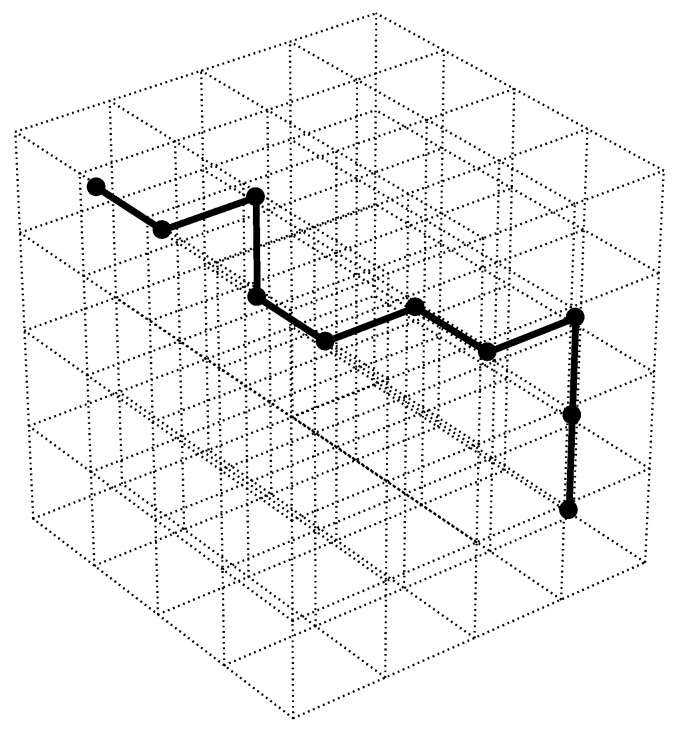
Visual representation of Equation (4) showing a three-dimensional lattice of a polymer solution mixture. $N_{0}$, total number of lattice sites (all cubes): $N_{s}$, number of solvent molecules (empty cubes); and $N_{p}r$, number of polymer repeat units (cubes with black dots connected with black lines) [30].

In order to derive Equation (9), referring to the three-dimensional lattice in Figure 2, the total number of interactions any given molecule within the lattice experiences is equal to six, assuming only interactions in the principal x, y, and z directions are considered. Therefore, the total number of contacts between a polymer lattice site and its polymer neighbors is z−4, where *z* is the total number of interactions (i.e., z=6). Given that the probability that a given molecule within the lattice is a solvent molecule is equal to the solvent volume fraction, the total number of polymer to solvent interactions ($N_{p,s}$) within the lattice is

(10) $$N_{p,s}=\left(z-4\right)rN_{p}\phi_{s}.$$

The change in energy for the formation of a polymer to solvent interaction ($\Delta \epsilon_{p,s}$) is defined as

(11) $$\Delta \epsilon_{p,s}=\epsilon_{p,s}-\frac{\left(\epsilon_{p,p}+\epsilon_{s,s}\right)}{2},$$

where the ϵ terms are the energies associated with polymer–solvent (p,s), polymer–polymer (p,p), and solvent–solvent (s,s) interactions. The change of internal energy associated with the enthalpy of mixing with the lattice ($\Delta U_{mix}$) can be defined as

(12) $$\Delta U_{mix}=\Delta \epsilon_{p,s}\left(z-4\right)rN_{p}\phi_{s}=\Delta \epsilon_{p,s}zN_{s}\phi_{p}.$$

Replacing $\Delta \epsilon_{p,s}$ with $k_{B}T\chi_{p,s}$ results in the final form of Equation (9). Subbing Equation (9) and Equation (5) into Equation (8) results in the Helmholtz free energy in the polymer solution:

(13) $$\Delta F_{mix}=k_{B}T\left[\frac{\phi_{p}}{rv_{r}}ln\left(\phi_{p}\right)+\frac{\phi_{s}}{v_{s}}ln\left(\phi_{s}\right)+\frac{\chi_{p,s}\phi_{s}\phi_{p}}{\sqrt{v_{r}v_{s}}}\right].$$

Differentiating Equation (13) with respect to the number of solvent molecules ($N_{s}$) results in the partial molar free energy associated with mixing (or the chemical potential), resulting in

(14) $$\Delta \mu_{s}=\frac{\partial \Delta F_{mix}}{\partial N_{s}}=RT\left[ln\left(1-\phi_{p}\right)+\left(1-\frac{1}{r}\right)\phi_{p}+\chi_{p,s}\phi_{p}^{2}\right].$$

Finally, utilising the relationship between the chemical potential and osmotic pressure, the mixing pressure can be calculated as

(15) $$\Pi =-\frac{\mu_{s}}{V_{s}}=-\frac{RT}{{\bar{V}}_{s}}\left[ln\left(1-\phi_{p}\right)+\left(1-\frac{1}{r}\right)\phi_{p}+\chi_{p,s}\phi_{p}^{2}\right],$$

where ${\bar{V}}_{s}$ is the molar volume of the solvent. Assuming infinite repeat units *r* within the polymer solution, the mixing pressure is simplified to

(16) $$\Pi_{mix}=-\frac{RT}{{\bar{V}}_{s}}\left[ln\left(1-\phi_{p}\right)+\phi_{p}+\chi_{p,s}\phi_{p}^{2}\right].$$

As the Flory–Huggins interaction parameter is dependent on both temperature and solvent composition (Equation (9)) and is normally calculated empirically, it is more appropriate to denote it by means of a series [31,32]:

(17) $$\chi_{p,s}=\chi_{0}+\chi_{1}\phi_{p}+\chi_{2}\phi_{p}^{2}+\ldots$$

This leads to the most common form of the Flory–Huggins mixing pressure equation,

(18) $$\Pi_{mix}=-\frac{RT}{{\bar{V}}_{s}}\left[ln\left(1-\phi_{p}\right)+\phi_{p}+\chi_{0}\phi_{p}^{2}+\chi_{1}\phi_{p}^{3}\right].$$

The above derivation of the mixing energy according to Flory–Huggins theory assumes molecular incompressibility (Equation (1)) and random distribution of the polymer and solvent lattice sites. Equation (18) can be implemented into both Flory–Rehner and mixture theories.

### 2.2. Ionic Energy

The ionic energy is most prominent in ionized hydrogels (hydrogels with an attached charge to the polymer chains) where there is a positive counter-ion concentration inside the gel, resulting in the generation of an osmotic pressure with the external solution. This external solution is assumed to be infinitely large, such that its concentration is prescribed and is kept at constant temperature and pressure. The osmotic pressure difference between inside and outside the gel drives fluid across the semi-permeable external membrane. Assuming that the translational entropy of the ions is the only contributor to the osmotic pressures, the ionic pressure is denoted as

(19) $$\Pi_{ion}=k_{B}T\sum_{i}\left(n_{i}^{in}-n_{i}^{out}\right),$$

where $n_{i}$ is the concentration of species *i* inside the gel (in) and in the external solution (out). The molar concentration ($c_{i}$) of each species can be calculated by

(20) $$c_{i}=\frac{n_{i}}{N_{A}}.$$

Subbing Equation (20) into Equation (19) for positive (+) and negative (−) ions results in the ionic pressure, in terms of molar concentration of the counter-ions and the ionic composition of the external solution:

(21) $$\Pi_{ion}=RT\left[\left(c_{+,in}+c_{-,in}\right)-\left(c_{+,out}+c_{-,out}\right)\right],$$

where *R* is the universal gas constant and the internal concentrations can be calculated from Donnan equilibrium:

(22) $$\left(c_{+,in}+c_{-,in}\right)=\sqrt{{\left(c_{CI}\right)}^{2}+4{\left(c_{ext}\right)}^{2}},$$

where $c_{CI}$ is the concentration of osmotically active counter-ions inside the gel. As mixture theory averages out the micro-structure, this description of the ionic energy is sufficient for continuum theories. However, when delving into the micro-structural effects on the ionic energy, alternative descriptions can be used [21]. Due to the difficulty of generating a correct model of the micro-structure of the material, as well as defining experimental values for those models (e.g., ionized groups per chain, number of monomer chains, electrostatic repulsion between chains, and so on), significant retro-engineering is necessary to describe the condition being assessed [24,33,34,35,36,37]. Although significant variation occurs between the models, even in non-equilibrium transient states of swelling, Donnan equilibrium is acceptable for gels, as the characteristic times of ionic diffusion, $h^{2}/D_{+}$ and $h^{2}/D_{-}$, is generally much smaller than the characteristic time of hydraulic pressure diffusion of the solvent, $h^{2}/KE$ (*h* = characteristic distance, *K* = hydraulic permeability, and *E* = Young’s modulus) [11]. This hints that Equation (19) is sufficient in describing the ionic pressure in gels for both Flory–Rehner and mixture theories.

### 2.3. Elastic Energy

According to Equation (3), the ionic and mixing energies are responsible for the swelling of the gel. In order to counteract this swelling and cease the deformation of the polymer chains, the elastic energy acts as the balancing force. How this energy is described is the biggest discrepancy between Flory–Rehner and mixture theories.

#### 2.3.1. Statistical Mechanics: Flory–Rehner Theory

The Flory–Rehner description of the elastic energy in a cross-linked network originates from the rubber elasticity theory of hyperelastic materials, which relies on two main assumptions: (i.) The free energy of the cross-linked network associated with its elastic response is the summation of the elastic energy of the individual chains within the network, and (ii.) the chains within the network follow a normal Gaussian distribution [30]. The aim of this statistical method to deduce the mechanics of the model is to relate the molecular structure of the gel network to its elastic macroscopic properties [38]. There are several important molecular characteristics that influence these elastic properties most. These characteristics include the concentration of elastic chains in the gel, the cross-link functionality, and network imperfections. As an in-depth review of the molecular characteristics of a gel network is outside the scope of this review, the reader is directed to the comprehensive review on this topic in [39]. The original definition for the change in the elastic free energy ($F_{elastic}$), according to Flory–Rehner, was taken as a function of the temperature and the change of entropy ($\Delta S_{d}$) within the system, with respect to its deformation:

(23) $$\Delta F_{elastic}=-T\Delta S_{d},$$

with the entropy of deformation derived as

(24) $$\Delta S_{d}=k_{B}N_{p}\left(\alpha^{2}+\frac{2}{\alpha }-3\right)/2,$$

where $N_{p}$ is the number of chains in the system and α is the deformation of the system, calculated as

(25) $$\alpha ={\left(\frac{\phi_{p,0}}{\phi_{p}}\right)}^{1/3}.$$

Since this original derivation, many statistical mechanics models for cross-linked networks have been proposed. These include phantom and network models (which only consider the influence of the individual network chains), as well as constrained junction fluctuation, diffused constraint, tube, and slip-tube models (which also take into account the interactions between chains). An in-depth review of each of these network models is outside the scope of this study, and more detailed information can be found in the vast literature on the subject [39,40,41,42,43].

However for the purpose of comparison to the continuum approach, the most basic of network model (the affine model) is described. The affine model assumes that the average displacements of the junctions (cross-link sites) transform affinely and, as a result, the elastic free energy is defined as

(26) $$F_{elastic(aff)}/k_{B}T=\left(\frac{N_{p}}{2V_{0}}\right)\left(\lambda_{1}^{2}+\lambda_{2}^{2}+\lambda_{3}^{2}\right)-\left(\mu_{el}/V_{0}\right)ln\left(\lambda_{1}\lambda_{2}\lambda_{3}\right),$$

where $k_{B}$ is the Boltzmann constant, *T* is the absolute temperature, $V_{0}$ is the original volume of the network, $\mu_{el}$ is the number of elastic junctions in the network, and $\lambda_{1,2,3}$ are the stretches per chain in the principal directions [44,45,46]. The stress in the system can, then, be related by differentiating the elastic free energy with respect to the principal stretches:

(27) $$\sigma_{eff(i)}=\frac{\lambda_{i}}{J}\frac{\partial F_{elastic(aff)}}{\partial \lambda_{i}},$$

where i=1,2,3, $\sigma_{eff(i)}$ denotes the effective stresses in the principal directions, and *J* denotes the volume change ($J=\lambda_{1}\lambda_{2}\lambda_{3}$).

Obviously, the limiting factor of this method of mechanically characterizing the elastic response of the solid matrix through statistical relations to molecular parameters is the calculation of these parameters. Combining the selection of mixing, ionic, and elastic energy equations results in a full description of the free energy within a swelling model. The application of these sets of equations to the coupling of deformation and fluid transport in hydrogels came several decades after the initial derivation of the Flory–Rehner theory [47].

Although the Flory–Rehner theory is well-defined and has been used in a plethora of studies, the difficulty of understanding the micro-structural deformation has led to a variety of models (as mentioned above). However, in order to relate the micro-structure back to the overall mechanical load, continuum mechanics has to be utilized. So, although Equation (26) defines the Gaussian-chain elastic response, relating the energy to gel swelling is completed through a continuum equation for the calculation of the effective stress, Equation (27). This has been present in several studies on the topic [17,48,49,50]. A large deformation model has been developed to include a Terzaghi decomposition of the stress in the system into the effective stress on the solid matrix and the pressure of the fluid in the pores [51,52,53,54]. This model was, then, applied to the free swelling of spherical hydrogels under osmotic loading [10]. During transient swelling, the deformation was shown to be strongly inhomogeneous, with the outer surface swelling first. The fluid volume fraction also increased at the surface, in order to coincide with the deformation gradient. The result of this gradient was the total azimuthal stress at the surface turning compressive, which has been strongly linked with the formation of surface instabilities.

Overall, Flory–Rehner theory utilizing statistical mechanics has shown an ample capability for describing the deformation seen in swelling hydrogels.

#### 2.3.2. Continuum Mechanics: Mixture Theory

Biot theory (also known as poroelasticity theory) lies at the core of continuum mechanics in porous solids and, therefore, a basic understanding of the approach is required. Originally derived for three-dimensional consolidation in soils [52], it has been altered to describe the non-linear finite deformation of fluid-saturated porous media [55]. The theory models the deformation of a solid with respect to fluid flow in the Lagrangian frame and vice versa. Biot has highlighted the importance of the Langrangian description, as the solid matrix controlling the translation and rotation of the adjacent pores (and, therefore, the material description) has proven to be essential. The deformation is defined by standard Finite Elasticity theory [56,57] as the deformation tensor (**F**), which is related to the strain through the right Cauchy–Green strain (**C**):

(28) $$F=\frac{\partial x}{\partial X},$$

(29) $$C=F^{T}F,$$

(30) J=det(F).

where *x* and *X* are the spatial coordinates in the deformed and reference state, respectively, and *J* is the Jacobian of the deformation tensor, denoting the volume change of the system with respect to the reference frame. The first and second Piola–Kirchhoff stresses are used as measures. First, the total stress in the system ($\sigma_{total}$) needs to be defined, and is split into the effective stress acting on the solid matrix ($\sigma_{eff}$) and the pressure on the fluid in the pores (*p*) through Terzaghi’s decomposition [54]. The total stress is symmetric and is the measure of stress in the Eulerian description:

(31) $$\sigma_{total}=\sigma_{eff}-pI.$$

The effective stress is calculated through the derivative of the selected strain energy density function ($\Delta F_{elastic}$), with respect to the right Cauchy–Green strain:

(32) $$\sigma_{eff}=J^{-1}F\frac{\delta \Delta F_{elastic}}{\delta C}F^{T}.$$

Popular strain energy density functions for hydrogel models are the Mooney–Rivlin and neo-Hookean models, due to their derivation spawning from polymer networks [56,58]. The first and second Piola–Kirchhoff stresses (**T** and **S**, respectively) are, then, given by

(33) $$T=J\sigma_{total}F^{-T},$$

(34) $$S=JF^{-1}\sigma_{total}F^{-T},$$

where **T** is non-symmetric and **S** is symmetric. Typically, the second Piola–Kirchhoff stress tensor is used in the Lagrangian description involving large deformations [59]. The pore pressure is equal to the summation of the chemical potential of the fluid inside the porous solid ($\mu_{f,gel}$) and the osmotic swelling pressure ($\Pi_{swelling}$ = $\Pi_{ionic}$ + $\Pi_{mixing}$):

(35) $$p=\mu_{f,gel}-\Pi_{swelling}.$$

Finally, the fluid flow (*Q*) inside the porous solid is described by a generalized Darcy’s law

(36) $$Q=-k\frac{\rho }{\nu }\frac{\partial \mu }{\partial x},$$

where *k* is the intrinsic permeability, ρ is the fluid specific mass, ν is the fluid viscosity, and the chemical potential μ derivative is the chemical gradient [60]. With the basic Lagrangian descriptions of the solid deformation and fluid flow described, mixture theory is then implemented. Originally derived for modeling any type of mixture [61], mixture theory has also been applied to modeling soft porous media [62]. It has also been applied to a variety of soft biological tissue and even cells [63,64,65]. The assumption made for mixture theory is that the continuum is occupied by all the constituents of the mixture at every point in the system. Biphasic, triphasic, and quadriphasic theories, taking into account the solid, fluid, and ionic components have all been published [66,67,68]. The novelty of mixture theory is not the capability to reach large deformations (this was already present in Biot theory) [55], but rather the ability to include ions into the theory. The positions of each constituent are described by

(37) $$x_{a}=\chi_{a}\left(X_{a},t\right),$$

where *a* is the constituent, *t* is time, $x_{a}$ are the position vectors in the current domain, $X_{a}$ are the position vectors in the original domain, and $\chi_{a}$ is a continuously differentiable mapping function from the original domain to the current domain. The deformation gradient of constituent, *a*, can be calculated by

(38) $$F_{a}={\left[\nabla_{a}⊗\chi_{a}\left(X_{a},t\right)\right]}^{T}.$$

Equation (38) allows the deformation of any constituent within the mixture to be calculated. Transformations between the deformed and reference states can be completed by multiplying the variable of interest by the determinant of the deformation tensor, with respect to the reference state (*J*) as a result of the mapping function ($\chi_{a}$) in Equations (37) and (38). For example, the original polymer volume fraction ($\phi_{p,0}$) within the mixture can be related to the current solid volume fraction ($\phi_{p}$) by

(39) $$\phi_{p,0}=J\phi_{p}.$$

The system is then governed by two equations describing the momentum balance (Equation (40)) and the fluid content continuity in the Lagrangian frame (Equation (41)) [53]:

(40) $$\nabla_{X}\cdot T=0,$$

(41) $$\frac{DJ}{Dt}+\nabla_{X}\cdot Q=0,$$

where **T** is the first Piola–Kirchoff stress tensor, $\nabla_{X}$ is a divergence operator applied to the Lagrangian variable *X*, and **Q** is a Lagrangian vector with its Eulerian counterpart as **q** = J$F^{-1}$**Q**.

In order to understand the historical development of the application of mixture theory, biological tissue studies also need to be considered. Initial models implementing this theory concentrated on quadri-phasic continuums in one-dimensional tissue swelling or the equilibrium swelling state [69,70,71]. The theory was, then, extrapolated to three dimensions in the large deformation regime [72]. However, local mass balance violation restricting the strain to approximately 10 percent and computational expense remained the main limitations.

The application of alternative finite element methods to mixture theory have also been published. Hong et al. [17] developed a theory of coupled diffusion and large deformation in polymeric gels and compared their results of uniaxial creep against Biot [52], published 70 years earlier. This theory expands on the pioneering work of a later Biot theory of finite deformation of porous solids [55], integrating aspects of Biot theory into Flory–Rehner theory. Since then, several non-linear swelling models have been presented based on the non-linear theory presented by Hong et al. [48,49,50,73]. Although the numerical model of Zhang et al. [48] showed stability in the formation of surface instabilities during transient swelling, the two-field formulation (network displacement and fluid chemical potential) can lead to inaccuracies [74]. A three-field mixed formulation, taking displacement, pore pressure, and fluid displacement, was derived to model hydrated biological tissue. With the addition of an extra independent variable, Lagrangian multipliers were used to constrain the system and significantly reduce computational expense [75]. Confined compression was used as the numerical example showing the advantages of the three-field formulation over the conventional two-field formulation. This three-field formulation was further developed and implemented into a three-dimensional free swelling model of a cube. The primary variables consisting of the displacement of the structure, the pore pressures, and the fluid velocities instead of the fluid displacements. Also, the model allows for the inclusion of a selection of hyperelastic strain energy density function constitutive laws [76].

Although these models capture finite deformation in the traditional sense of its meaning, the finite deformation in hydrogel swelling should be seen as much larger than conventional materials. In order to model large deformations in soft materials, the Mixed Hybrid Finite Element Method (MHFEM) has shown promising results. This technique uses a hybridization of the mixed finite element method to reduce the degrees of freedom in the system, hence considerably decreasing computation time. During conventional modeling of hydrogels using standard FEM techniques, the fluid flux of the system is calculated through numerical differentiation of the chemical potential field [74]. This can lead to inaccuracies in defining the fluid flux field, which is a critical variable in describing the fluid–solid coupled deformation. Therefore, calculating the fluid flux as a primary variable through Raviart–Thomas element ensures local mass conservation and has been proven to be more effective in solving Darcy-type equations [77,78]. This is especially true for processes with an inhomogeneous permeability tensor. As finite swelling of gels typically leads to large variation of the hydraulic permeability across the domain, MHFEM has proved to be far superior to classical FE formulations. Initially, MHFEM has been implemented to solve Biot consolidation problems concentrated on ground water in geomechanics, showing increased simulation accuracy [79,80]. Its success has led to its implementation in swelling models. Solving for the displacement, chemical potential, and fluid flux as primary variables, the swelling of tissue and hydrogel has been modeled for small deformations in two dimensions using a bi-phasic theory [81]. This model was extended to three dimensions for the free swelling and shrinking of gels [11]. A strain-dependent hydraulic permeability factor was also added, allowing for more control of the dynamic swelling behavior. The results showed the ability to deform the gel up to 80 times its original volume, while allowing a stiffness approaching the visco-elastic fluid range (<5 kPa). Also, the enhanced local mass conservation allowed for irregular gel geometries to be modeled, as well as simulating the formation of surface wrinkles under surface-attached swelling. These were, then, verified against experimental tests on the same phenomenon, further displaying the robustness of the model [82]. Table 1 summarizes the computational models reviewed in this section.

## 3. Experimental Analysis

### 3.1. Deformation Measurements

The three-dimensional mechanical characterization of hydrogels under osmotic loading is a challenging task. For many computational swelling studies, experimental verification of the model is conducted by comparing the volume change ratio of the equilibrium state. Although this is sufficient for validating the equilibrium states, a comparison of the transience of the swelling is often ignored. For many applications, mainly drug delivery, the transience of the system is of great importance in understanding the characteristics of the delivery mechanism. Therefore, this section presents the current literature surrounding the experimental quantification of hydrogel swelling.

The most important characteristic of hydrogel swelling is the volume change, also referred to as the volumetric deformation, which is measured as a ratio of the volume before swelling to the volume after swelling. Volume change can be experimentally calculated by two separate methods, depending on the geometry of the gel. Firstly, if the gel is of a regular geometry, the geometrical features (radius, height, width, and so on) before and after swelling can be measured (by physical measurement or by imaging). For example, radial measurements can be used to calculate the volume change (ΔV) from

(42) $$\Delta V=\frac{\frac{4}{3}\pi {\left(r_{after}\right)}^{3}}{\frac{4}{3}\pi {\left(r_{before}\right)}^{3}},$$

where $R_{after}$ and $R_{before}$ are the radii of the gel after and before swelling, respectively. The second technique to experimentally calculate volume change utilizes the mass change of the gel (also known as the capacity). The capacity can be calculated as

(43) $$C=\frac{M_{s}}{M_{g}}=\frac{M_{f}+M_{g}}{M_{g}},$$

where $M_{g}$ is the mass of the dry polymer, $M_{s}$ is the mass measured after swelling, and $M_{f}$ is the mass of the fluid. However, the capacity measurement does not take into account the porosity of the initial state.Therefore, the volume of air in the initial state needs to be considered. Additionally, the mass readings must be adjusted by density to provide a volume change measurement. The volume change can be written as

(44) $$\Delta V=\frac{V_{after}}{V_{before}}=\frac{V_{fluid}+V_{gel}}{V_{gel}+V_{air}},$$

where $V_{fluid}$, $V_{gel}$, and $V_{air}$ are the volumes of fluid, gel, and air respectively. Re-writing the volumes in terms of mass and density,

(45) $$\Delta V=\frac{\frac{m_{fluid}}{\rho_{fluid}}+\frac{m_{gel}}{\rho_{gel}}}{\frac{m_{gel}}{\rho_{gel}}+V_{air}},$$

where $m_{fluid}$ and $m_{gel}$ are the masses of the fluid and gel and $\rho_{fluid}$ and $\rho_{gel}$ are the densities of the fluid and gel, respectively. The volume of air in the dry state is a function of the mass and porosity of the dry gel:

(46) $$V_{air}=\frac{m_{gel}}{\rho_{gel}}\left(\frac{\phi }{1-\phi }\right),$$

where ϕ is the porosity of the dry gel (the volume fraction of air). Therefore, the volume change can be calculated from capacity measurements. However, if the size of a hydrogel is very small, an accurate measurement of the dry state mass is difficult. Micro weighing scales can be used, but the error in the measurement increases significantly. Furthermore, due to the difficulty of measuring the porosity and effective density of a dry gel, the conversion from capacity to volume change also produces significant experimental error. Therefore, a relationship between the swelling ratio of a group of small gel beads and a single gel bead has been developed, taking into account the interstitial fluid between the gels [86,87]. This technique utilizes the radius of the gel and, therefore, is only applicable to approximately spherical geometries. For more irregular shapes, a centrifuge retention capacity (CRC) method can be used, which averages the capacity across a material sample; for example, of 0.2 g (i.e., a multitude of individual particles) [88]. The interstitial fluid is removed by centrifuge and the remaining hydrogel is measured and compared to the initial state, as per Equation (43). Only accounting for equilibrium states, this capacity calculation has also been used to create a discrete field of transient swelling [47]. However, it is only useful for slow-swelling hydrogels with small deformations, as increasing the swelling speed and magnitude results in a subsequent increase in swelling variance at each time step.

### 3.2. Mechanical Behavior

During standard mechanical characterisation, the stresses and strains of the material are recorded and a corresponding elastic or shear modulus is calculated. During osmotic loading, the stress in the system cannot be recorded in-situ as mechanical clamps are not feasible for free swelling. The stress could potentially be determined through a constitutive relation implemented after conducting the experiments. However, the constitutive model would have to be assured to replicate the stress-strain behavior of the material. Therefore, when studying osmotic loading experimentally, it is wise to stick to the strains and modulus of the system. Several methods exist to measure the modulus of the material. Firstly, confined compression restricts the swelling of a soft material in one dimension and measures the associated stresses acting on the confinement piston [89]. Secondly, nano-indentation can be used at various swelling ratios and can deduce a dynamic stiffness as a function of volume change [90,91]. Finally, a oscillatory shear method can be implemented to calculate the shear modulus of gel discs and, again, gather a relationship between stiffness and volume change [92].

**Figure 3:**
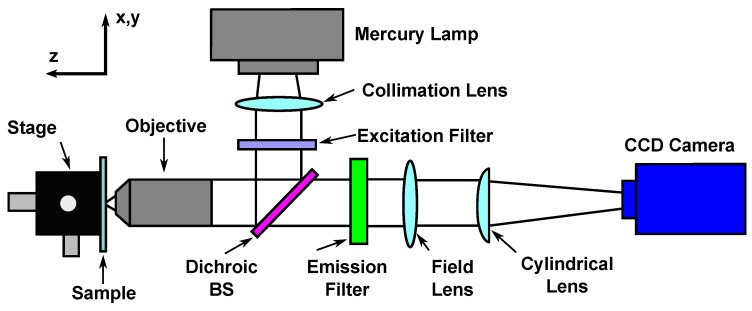
Schematic of a particle tracking velocimetry (PTV) setup used to quantify the transient swelling of hydrogels in three dimensions [97]; adapted from [96]. Placing a cylindrical lens in front of the CCD (charge-coupled device) camera creates an anamorphic imaging system, resulting in different optical properties in the x and y directions. Combining the x and y projections allows for the information of the z-dimension to be calculated [102]. (BS = beamsplitter)

As mechanical clamping is not feasible for osmotic loading, in relation to the measurement of strain, digital image correlation (DIC) is the logical choice of methodology. However, the swelling of a hydrogel can span a full order of magnitude of displacement. Therefore, attaining images in three dimensions that couple high spatial resolution with a large field of view and high temporal resolution is difficult. Several studies have implemented a standard camera setup to capture the change in gel radius over time. This is beneficial for calculating an approximation of the volume change and gathering a basic understanding of the transient swelling behavior of spherical geometries [10,93]. Three-dimensional techniques have been implemented using confocal microscopy [94,95]. Yet, as confocal imaging in 3D requires stacks of images to be taken, the temporal resolution is too low for such a highly dynamic process. More recently, transient swelling in three dimensions was attempted using an altered particle tracking velocimetry (PTV) setup which incorporated wave-front sensing (Figure 3) to allow a z dimension to be calculated with only one camera [96,97]. Fluorescent micro-particles were embedded inside the hydrogel from the polymerization stage, and these were tracked. Although the system captured a z-dimension, it was restricted to 200 μm and, therefore, the majority of the swelling was outside the field of view. Nonetheless, this study provided the framework for future studies on capturing the transient swelling of hydrogels. Furthermore, advances of Dynamic Light Scattering (DLS) used to probe the diffusion of particles has allowed for the quantification of the transient properties of polymer chains at a microscopic level, providing a better understanding of the rheology of the system [98,99]. With recent advances in imaging capabilities, mostly as a result of the need for CMOS image sensors in the mobile phone industry, there exist several potential systems for this application [100,101].

## 4. Transient Surface Instabilities

Surface instabilities occur in most soft materials, including hydrogels, as well as in biological tissue. The low moduli, sensitivity to environmental stimuli, and large deformation of these materials results in the buckling of their external surfaces. Conventionally, buckling is seen as a failure mode of a material. However, for soft materials, the ability to control the surface buckling effects during transient swelling has led to some interesting applications, such as use in microfluidic devices, sensors, smart adhesives, and the control of cellular behavior in tissue engineering [103,104,105]. As a result, the understanding of the mechanisms driving this phenomenon is essential in controlling the effect. Many studies, both theoretical and experimental, have been published on this topic. Several of these studies have concentrated on surface-attached hydrogel layers exhibiting surface instabilities [73,106,107,108,109]. However, this section reviews the current theoretical and experimental literature surrounding surface instabilities forming on spherical or spherical-like geometries.

**Figure 4:**
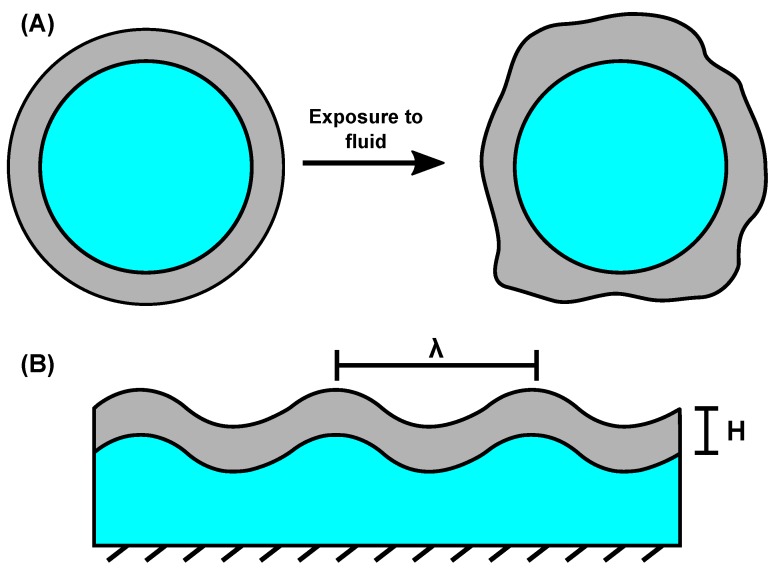
(**A**) A circular core (blue)–shell (grey) hydrogel, before and after exposure to a swelling solution, showing the anisotropy in the buckling formation. (**B**) Surface-attached hydrogel showing the wavelength, λ, and layer thickness (H).

The morphological buckling response of soft materials can be categorized into three phenotypes: Wrinkling, folding, and creasing [110]. Wrinkling is defined as periodic surface fluctuations formed on an originally smooth surface, folding as deep localized valleys, and creasing as self-contacting ridges. Each of these are a consequence of an elastic surface layer which is free to swell around an attached stiffer core, or vice versa [111]. This mechanical constraint is created when fluid diffuses into a gel, but has not yet permeated to the core. Compressive surface stresses cause the surface to buckle. The magnitude of the buckling (and, hence, the phenotype) is dependent on the magnitude of the compressive surface stress, as well as the wavelength of the pattern (denoted as λ in Figure 4B). Both of these are also dependent on the thickness of the buckling layer (also denoted in Figure 4B, as H) [11,111].

As a modulus difference is required for the formation of surface buckling, many studies have coupled a stiff gel to a one that is softer. In two dimensions, the effect of the elastic modulus ratio (θ) between the core and surface (Equation (47)) has been experimentally assessed [112]:

(47) $$\theta =\frac{G_{1}}{G_{2}},$$

where $G_{1}$ and $G_{2}$ are the moduli of the core and shell, respectively. For ratios less than 1 (i.e., the surface is stiffer than the core), the resulting wavelength was large, causing big lobes to form on the surface of the gel. On the other hand, larger modulus ratios (i.e., a stiffer core with an elastic surface) caused small instabilities, replicating a wrinkling pattern. The coupled investigation of swelling kinetics and instability formation was conducted using Nuclear Magnetic Resonance (NMR). NMR contrasts the swollen regions of a swelling gel from the unswollen regions through spin-lattice rates and various contrast agents [113]. As a result, qualitative evidence of the swelling surface buckling was presented, showing the importance of the modulus ratio (Equation (47)). Furthermore, as the thickness of the swelling surface decreases, the wavelength of the buckling increases accordingly.

The ratio of moduli has been continued in computational studies and extended to three dimensions. From investigating gyrification effects on the brain, the formation of sulci was shown to be strongly dependent on the mechanical constraint created by the surface grey matter swelling at different rates to the core white matter [114]. Both two- and three-dimensional simulations were conducted, with the ‘core’ being clamped to a non-deforming centre. Similar moduli for the surface and core proved significant in the formation of the correct brain morphology. A similar study extended this theory by experimental verification of the model [115]. From Magnetic Resonance Images (MRI) images of a fetal brain, a 3D-printed gel mimic was created and placed in a swelling solution. The mechanical swelling was alone sufficient in replicating the sulci of the brain and showed remarkable similarity to both MRI images of real brains at similar time points and the three-dimensional numerical model. These results showed the importance of mechanical constraints over bio-molecular determinants in correct geometrical fetal brain formation.

More recently, studies have moved towards showing instability formation in the unconstrained swelling of gels. Three-dimensional surface instabilities were simulated with a constant modulus across the radius through a stress-diffusion model [116]. The effects of the shear modulus and the dimensionless Flory–Huggins parameter, χ, on the formation of surface instabilities have been investigated, where the lowest modulus and Flory parameter (meaning more pronounced swelling) resulted in the largest buckling of the surface layer. The model showed that the hoop stress at the surface of the gel was in compression, whereas the core was in tension. However, further investigation is required to quantify the effects of many other parameters, such as permeability, geometry, porosity, fixed charge, and external concentration on surface wrinkling. Therefore, although a handful of studies have successfully experimentally and numerically replicated surface instabilities in soft materials, the characteristics which govern the process remain under-studied.

## 5. Conclusions

As discussed, the swelling of hydrogels is a multivariate, complex process. Many studies have been published on the computational modeling of this phenomenon, mostly centered around theoretical frameworks of Flory–Rehner, Biot, and mixture theories. The basics of the frameworks, including their similarities and differences, have been presented here, with the accompanying studies implementing them. As shown, the theories have been used to generate powerful swelling models for large deformations. However, no study has shown thorough transient experimental deformation verification and, therefore, it is difficult to hypothesize which of them is more representative. This highlights the importance of experimentally quantifying transient and local swelling in hydrogels, in order to design the most effective solutions for drug delivery and tissue engineering systems. The lack of methodologies for quantifying the transient state of swelling has led to a dearth of experimental validation for these numerical models. With recent advancements in technology, more and more potential systems have become available that can be applied for this purpose. Finally, the current state-of-the-art regarding surface buckling was described. Although much advancement in the understanding of this phenomenon has taken place recently, there still exists a gap in the knowledge of the relative magnitudes of the contributions of various parameters. Overall, despite the myriad studies on numerical and experimental deformations of hydrogels, there still remains a lack of knowledge on the transient state of the process.

## Figures and Tables

**Table 1:** Computational models implemented to replicate deformation in porous structures defining the material, strain energy density function theoretical framework, and the dimension reached in each study.

Study	Material	Framework	Dimension	Reference
Tanaka and Fillmore 1979	Hydrogel	Statistical	2D	[47]
Bowen 1980	Hydrogel	Continuum	2D	[62]
Lanir 1987	Biological Tissue	Continuum	2D	[66]
Lai et al., 1991	Articular Cartilage	Continuum	2D	[67]
Huyghe and Janssen 1997	Porous Media	Continuum	2D	[68]
Oh et al., 1998	Hydrogel	Statistical	2D	[83]
Van Loon et al., 2003	Biological Tissue	Continuum	3D	[72]
Dolbow et al., 2005	Hydrogel	Statistical	2D	[84]
Malakpoor et al., 2007	Articular Cartilage	Continuum	2D	[81]
Hong et al., 2008	Hydrogel	Statistical	2D	[17]
Hong et al., 2009	Hydrogel	Statistical	2D	[70]
Kang and Huang 2010	Hydrogel	Continuum	2D	[71]
Chester and Anand 2010	Hydrogel	Statistical	2D	[49]
Duda et al., 2010	Hydrogel	Statistical	2D	[50]
Bouklas et al., 2012	Hydrogel	Statistical	2D	[85]
Bouklas et al., 2015	Hydrogel	Statistical	2D	[73]
Bertrand et al., 2016	SAP	Statistical	3D	[10]
Yu et al., 2018	SAP	Continuum	3D	[11]

## Abbreviations

The following abbreviations are used in this manuscript:

2D	Two-Dimensional
3D	Three-Dimensional
CCD	Charged Couple Device
CMOS	Complementary Metal Oxide Semiconductor
CRC	Centrifuge Retention Capacity
DIC	Digital Image Correlation
FEM	Finite Element Method
MHFEM	Mixed Hybrid Finite Element Method
MRI	Magnetic Resonance Imaging
NMR	Nuclear Magnetic Resonance
PTV	Particle Tracking Velocimetry
SAP	Superabsorbent Polymer
− (subscript)	Negative Charge
+ (subscript)	Positive Charge
$N_{a}$	Avagadro’s Number
$k_{B}$	Boltzmann Constant
C	Capacity
Δϵ	Change in energy for the formation of a polymer–solvent interaction
μ	Chemical Potential
n and c	Concentration (amount-of-substance and molar)
α (subscript)	Constituent
CI	Counter-ion

α	Deformation of the system (Statistical Mechanics)
F	Deformation Tensor
ρ	Density
*∇*	Divergence operator
S	Entropy
**T**	First Piola–Kirchhoff stress tensor
χ	Flory–Huggins Parameter
Q	Fluid Flux
ρ	fluid specific mass
G	Gibbs Free Energy
F	Helmholtz Free Energy
**I**	Identity Tensor
U	Internal Energy associated with Enthalpy
J	Determinant of the deformation tensor (Volume change)
**Q**	Lagrangian vector
λ	Principal stretch
χ	Mixture theory mapping function
M	Mass
*G*	Moduli
$\bar{\mu }$	Molar chemical potential
$\bar{V}$	Molar Volume
$\mu_{el}$	Number of elastic junctions
z	Number of lattice connections
N	Number of molecules
Π	Osmotic pressure
k	Permeability
p (subscript)	polymer
p	pore pressure
r	Radius
r	repeat units
**C**	right Cauchy–Green strain tensor
**S**	second Piola–Kirchhoff stress tensor
s (subscript)	solvent
σ	Stress
T	Temperature
R	Universal Gas Constant
V	Volume
ϕ	Volume Fraction
